# Oraler Lichen planus bei einer mit monoklonalen Anti‐CD20‐Antikörpern behandelten Patientin

**DOI:** 10.1111/ddg.15777_g

**Published:** 2025-10-23

**Authors:** Willy Chan, Alexander Nast, Gabriela Poch, Kamran Ghoreschi

**Affiliations:** ^1^ Klinik für Dermatologie Venerologie und Allergologie Charité – Universitätsmedizin Berlin corporate member of Universität Berlin and Humboldt‐Universität zu Berlin Berlin Deutschland

Sehr geehrte Herausgeber,

Eine 53‐jährige Patientin stellte sich in unserer dermatologischen Ambulanz mit schmerzhaften, bilateralen, weißlichen, retikulären Läsionen an der bukkalen Mundschleimhaut vor, im Sinne einer Wickham‐Streifung (Abbildung 1). Die Patientin klagte außerdem über Juckreiz und erhöhte Empfindlichkeit der Genitalschleimhaut sowie über Juckreiz und Schuppung der Kopfhaut. Die Genitalschleimhaut war frei von Hautveränderungen, die Kopfhaut zeigte flache schuppende Plaques. Die Nägel waren nicht betroffen. Die Familienanamnese war positiv für Psoriasis. Vor dem Auftreten der Hautsymptome hatte die Patientin eine Therapie mit Ocrelizumab, einem monoklonalen Anti‐CD20‐Antikörper, zur Behandlung ihrer multiplen Sklerose erhalten. Ocrelizumab (600 mg) war in Abständen von 6 Monaten verabreicht worden (Juni 2022, Dezember 2022, Juli 2023). Die ersten Hautsymptome traten 4 Wochen nach der dritten Infusion auf.

**ABBILDUNG 1:**
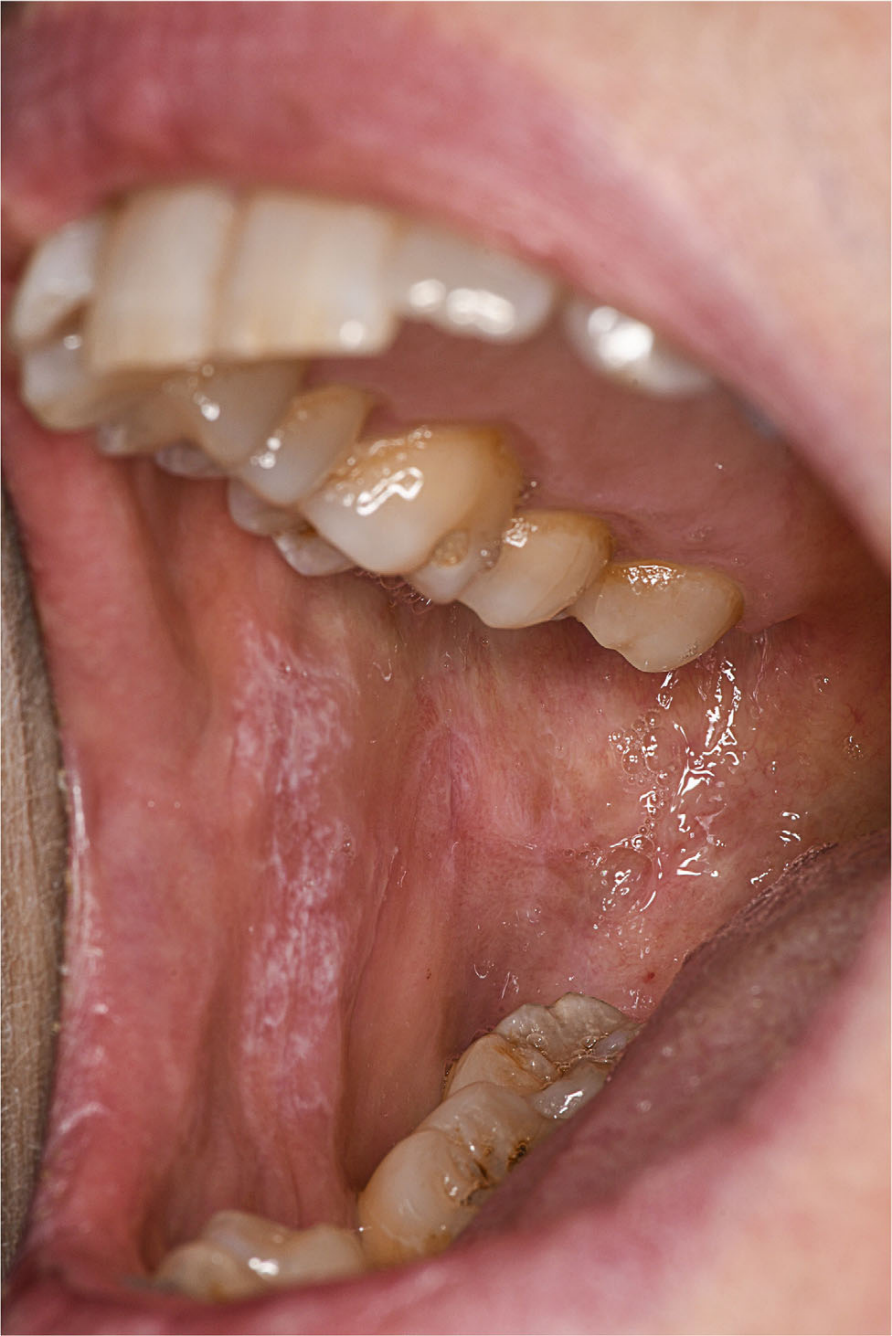
Weiße, netzartige Läsionen an der oralen, bukkalen Schleimhaut, im Sinne einer Wickham‐Streifung.

Wir führten eine Stanzbiopsie aus der Wangenschleimhaut durch. Die histopathologische Untersuchung zeigte ein bandförmiges lymphozytäres Infiltrat am dermoepidermalen Übergang mit Basalzellvakuolisierung im Sinne einer Interface‐Dermatitis, typisch für einen Lichen planus.

Die Ocrelizumab‐Infusionen wurden abgesetzt. Zur Behandlung des Lichen planus wurde eine topische Behandlung mit Glukokortikosteroiden eingeleitet: Eine Betamethason‐haltige Mundspüllösung wurde für die oralen Läsionen verwendet, eine Methylprednisolon‐Creme im Bereich der Genitalschleimhaut und ein Clobetasol‐Shampoo für die Kopfhaut. Mit diesem Therapieschema konnten die Hauterscheinungen deutlich reduziert werden, eine vollständige Remission wurde jedoch bisher nicht erreicht. Was die multiple Sklerose betrifft, befindet sich die Patientin derzeit in einem stabilen Zustand und erhält keine Behandlung. Eine Fortsetzung der Behandlung mit Anti‐CD20‐Antikörpern ist nicht geplant. Zu den künftigen therapeutischen Optionen gehören Dimethylfumarat oder Sphingosin‐1‐Phosphat‐Rezeptormodulatoren.

Um eine mögliche Kausalität oder Koinzidenz zwischen der Anti‐CD20‐Therapie und dem Auftreten eines Lichen planus zu untersuchen, recherchierten wir nach bereits veröffentlichten Fällen. Lediglich über einen Fall wurde in der Literatur berichtet, in dem eine durch eine Ocrelizumab‐Therapie verursachte lichenoide Reaktion beschrieben wurde.^1^ Zwei weitere Fälle konnten identifiziert werden, die mit den Anti‐CD20‐Antikörpern Rituximab^2^, ^3^ in Verbindung gebracht wurden sowie ein Fall mit Obinutuzumab.^4^ Im Gegensatz zu unserer Patientin zeigte sich in allen berichteten Fällen eine vollständige Remission nach topischen Kortikosteroiden und nach Absetzen der Anti‐CD20‐Therapie. Neben lichenoiden Reaktionen wurde auch über psoriasiforme Läsionen (vorwiegend palmoplantare Pustulose) im Zusammenhang mit einer Anti‐CD20‐Therapie berichtet.^5^, ^6^, ^7^, ^8^, ^9^ Da sowohl ein Lichen planus als auch eine Psoriasis T‐Zell‐vermittelte Erkrankungen darstellen, wurde eine hyperaktive T‐Zell‐Reaktion aufgrund der B‐Zellen‐Depletion vermutet, wobei der Pathomechanismus noch nicht vollständig geklärt ist.^3^ Interessanterweise gibt es auch Berichte über die erfolgreiche Behandlung eines Lichen planus durch Rituximab.^10^, ^11^, ^12^ Während lichenoide Reaktionen im Rahmen von unerwünschten Arzneimittelreaktionen häufiger auftreten, wurde sie selten in einem Zusammenhang mit Anti‐CD20‐Therapien beschrieben. Angesichts der großen Anzahl von Patienten, die in den letzten drei Jahrzehnten mit Anti‐CD20‐Antikörpern behandelt wurden, unterstreichen die insgesamt fünf veröffentlichten Fälle die Seltenheit dieser Nebenwirkung. Mit dieser Publikation möchten wir weitere Aufmerksamkeit auf Anti‐CD20‐Antikörper als mögliche Auslöser lichenoider Entzündungen lenken.

## DANKSAGUNG

Open access Veröffentlichung ermöglicht und organisiert durch Projekt DEAL.

## INTERESSENKONFLIKT

Keiner.
